# Shifting care from hospital to community, a strategy to integrate care in Singapore: process evaluation of implementation fidelity

**DOI:** 10.1186/s12913-020-05263-w

**Published:** 2020-05-24

**Authors:** Milawaty Nurjono, Pami Shrestha, Ian Yi Han Ang, Farah Shiraz, Ke Xin Eh, Sue-Anne Ee Shiow Toh, Hubertus Johannes Maria Vrijhoef

**Affiliations:** 1 Health Services Research, Changi General Hospital, Singapore, Singapore; 2 Saw Swee Hock School of Public Health, National University of Singapore, Singapore, Singapore; 3 Regional Health System Office, National University Health System, Singapore, Singapore; 4 Department of Patient and Care, University Hospital Maastricht, Maastricht, the Netherlands; 5 Panaxea B.V., Amsterdam, the Netherlands

**Keywords:** Integrated care, Shift from hospital to community, Multi-morbidity, PCMH, Process evaluation, Implementation fidelity, Realist evaluation

## Abstract

**Background:**

Accessibility to efficient and person-centered healthcare delivery drives healthcare transformation in many countries. In Singapore, specialist outpatient clinics (SOCs) are commonly congested due to increasing demands for chronic care. To improve this situation, the National University Health System (NUHS) Regional Health System (RHS) started an integrated care initiative,the Right-Site Care (RSC) program in 2014. Through collaborations between SOCs at the National University Hospital and primary and community care (PCC) clinics in the western region of the county, the program was designed to facilitate timely discharge and appropriate transition of patients, who no longer required specialist care, to the community. The aim of this study was to evaluate the implementation fidelity of the NUHS RHS RSC program using the modified Conceptual Framework for Implementation Fidelity (CFIF), at three distinct levels; providers, organizational, and system levels to explain outcomes of the program and to inform further development of (similar) programs.

**Methods:**

A convergent parallel mixed methods study using the realist evaluation approach was used. Data were collected between 2016 and 2018 through non-participatory observations, reviews of medical records and program database, together with semi-structured interviews with healthcare providers. Triangulation of data streams was applied guided by the modified CFIF.

**Results:**

Our findings showed four out of six program components were implemented with low level of fidelity, and 9112 suitable patients were referred to the program while 3032 (33.3%) declined to be enrolled. Moderating factors found to influence fidelity included: (i) complexity of program, (ii) evolving providers’ responsiveness, (iii) facilitation through synergistic partnership, training of PCC providers by specialists and supportive structures: care coordinators, guiding protocols, shared electronic medical record and shared pharmacy, (iv) lack of organization reinforcement, and (v) mismatch between program goals, healthcare financing and providers’ reimbursement.

**Conclusion:**

Functional integration alone is insufficient for a successful right-site care program implementation. Improvement in relationships between providers, organizations, and patients are also warranted for further development of the program.

## Background

Worldwide, rising prevalence of multi-morbidity among rapidly aging population increases demands for healthcare services and exert surmount pressures on healthcare systems. Disease-centric provision of healthcare is becoming inadequate and unsustainable in the longer terms [1]. There is thus an urgency to shift to people-centric provision of healthcare. Integrated care supports people-centered care through coordinating services around a person’s needs to improve outcomes, quality and affordability, especially for those with multiple chronic illnesses [2].

The World Health Organization (WHO) identified care continuity and care coordination to be essential in provision of integrated people-centered care [3] and advocates for a greater collaboration between hospitals and primary care [4]. Integrated chronic care programs led by hospitals were shown to result in positive health outcomes and patient experiences [4]. Likewise, transfer of healthcare services from hospitals to primary care, relocation of hospital services to primary care, and shared chronic care between primary care and hospitals were found to improve access to specialist services and reduce demands on acute hospitals [5, 6] and depression outcomes [7].

Similar to other developed countries, Singapore is facing the challenge of ensuring good quality and affordable healthcare services for its rapidly aging population and increasing prevalence of multi-morbidity [8]. In 2013, the early period of the Regional Health System (RHS), dyslipidemia, hypertension, diabetes mellitus, chronic kidney disease and coronary heart disease were found to be the top five chronic diseases seen across the public healthcare sectors [9].

Historically, Singapore’s healthcare system was designed with an emphasis on providing episodic care within acute hospitals in a largely disease-centric manner [10, 11]. While it has been ranked highly in terms of its healthcare system efficiency [12], Singapore was considered a low primary care country because of its system and practice characteristics [13] . Unlike other developed countries in which long-term chronic care is typically provided by primary care practitioners within the community, a high proportion of chronic long-term care is delivered by specialist outpatient clinics (SOCs) within the hospitals in Singapore [10]. As a result, SOCs are generally congested‚ resulting in long waiting time. Preference among the general public for hospital over primary care due to high financial subsidies in the hospitals and perceived low status of primary care has also kept the SOCs busy [5, 6].

In response to these pressures, the (RHSs) were established in Singapore to consolidate resources and work towards the common goal of providing integrated people-centered care close to where people live [8]. Led by a major public hospital, each RHS comprises acute and community hospitals, public primary care clinics (polyclinics), general practitioners, nursing homes, and other social care providers within the same geographical region [14].

In 2013, within the National University Health System (NUHS) RHS in Singapore, there were over 600,000 unique attendances at the SOCs in its primary acute hospital, National University Hospital (NUH). This was a significant rise from just under 500,000 unique attendances in 2009. In response for this rapid increase in SOC utilization, the National University Health System (NUHS) RHS initiated the Right-Site Care (RSC) program. Through collaborations between SOCs at the National University Hospital (NUH) and primary and community care (PCC) clinics within the western region of Singapore, the RSC program was designed to facilitate timely discharge and support appropriate transition from hospital to the community through care consolidation and improved care continuity.

**Fig. 1:**
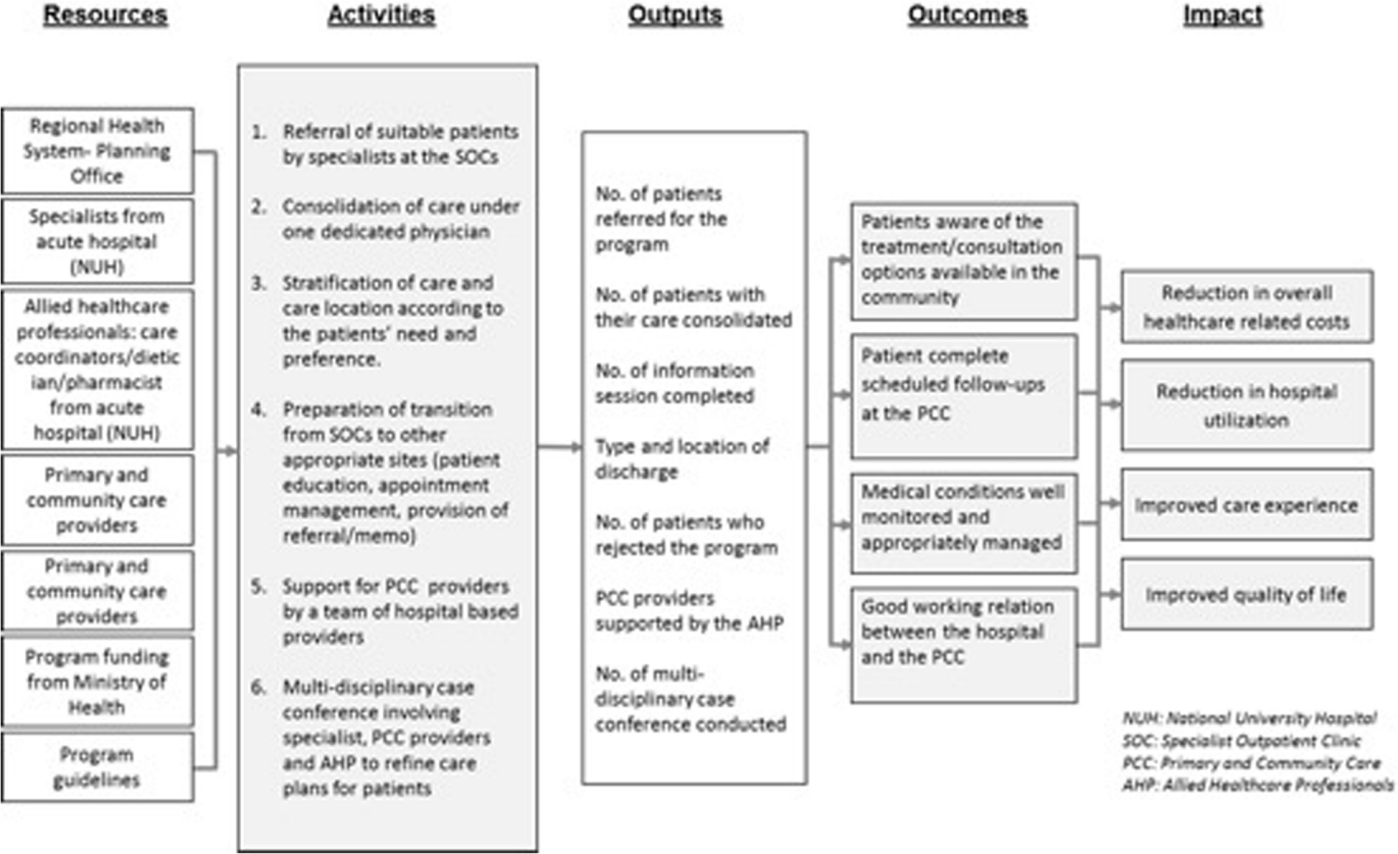
The logic model of NUHS-RHS Right-Site Care Program

Modelled after Patient Centered Medical Home (PCMH) practices in the United States of America (USA) [15] and the Netherlands’ Primary Care Plus Model [16], the RSC program aimed to: (i) reduce hospital utilizations, (ii) maintain quality of care, (iii) improve patients’ satisfaction, and (iv) reduce healthcare related cost. Figure 1 illustrates how the RSC program was expected to work. Consisted of 6 components which was developed and implemented simultaneously, the program pulls together various resources from across care settings to achieve program intended outcomes.

While clinical studies have been useful in discerning success of a program in achieving its intended outcomes, they are limited in their ability to inform insights on how a program can be improved [17]. Thus, there has been increasing emphasis to understand how complex programs, such as the RSC program, have been carried out. One way to achieve this is by assessing implementation fidelity – the degree in which a program is implemented as intended. It acts as a potential mediator of the relationship between programs, and their intended outcomes [18]. Higher implementation fidelity is associated with increased likelihood of success, and assessing implementation fidelity allows the true effects and reasons behind the success or failure of a program to be explicitly determined [18].

This study aimed to conduct process evaluation to examine the implementation fidelity of the NUHS RHS RSC program at providers, organizational, and system levels. In the context of Singapore, most prior evaluations of similar programs focused mainly on assessing the effectiveness with limited information on the implementation processes [19, 20], making it difficult to make program improvements. This study considered the importance of system level approaches in the implementation of a RSC program [21] and aimed to provide insights to explain the outcomes of an early cohort of the program reported by Ang et al. [22]. Also, this study sought to address the existing research gap where reports of similar initiatives in Singapore were largely commentaries [21, 23] and complement insights on patient experience [24]. As this gap also exist in other countries, results gathered from this study are anticipated to inform further development of similar programs beyond Singapore.

## Methods

### Study design

**Fig. 2:**
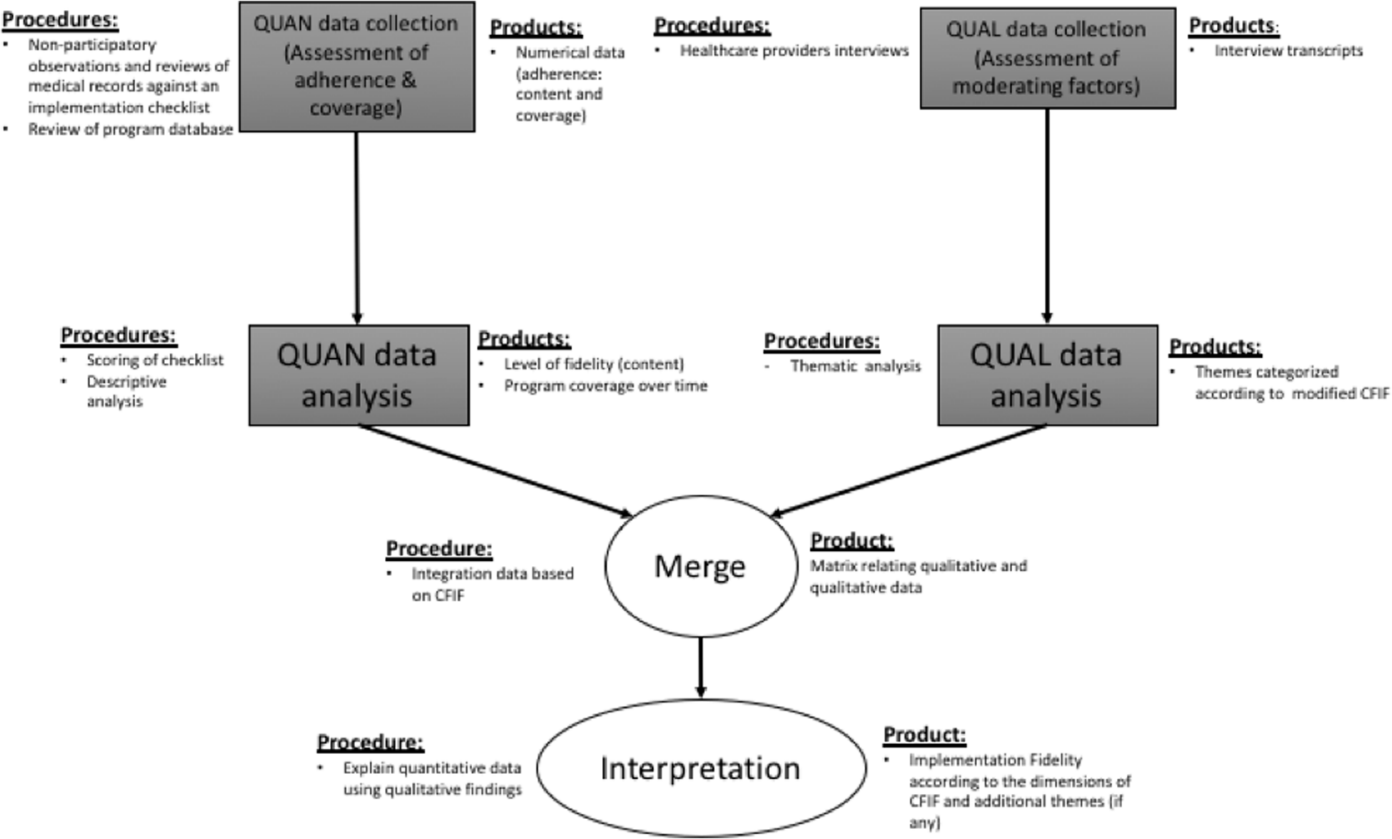
A convergent parallel mixed methods study to examine the implementation fidelity of NUHS-RHS Right Site Care Program

This study is a part of realist evaluation of the NUHS RHS [25]. Given the complexity of the RSC program, the range of perspectives in which this study tries to capture which could not be sufficiently assessed using solely quantitative or qualitative method, a convergent parallel mixed methods study (Fig. 2) was conducted. Both quantitative and qualitative were collected concurrently and given equal weightage.

### Setting and recruitment

The study was conducted between 2016 and 2018 in the NUHS RHS. The NUHS RHS has been tasked to provide care to approximately 1.1 million individuals who reside in the western part of Singapore. As of 2013, a total of 148,272 patients under NUHS RHS purview was found to have one or more chronic illnesses [9]. Study participants were recruited from various collaborative units of the program including NUH and PCC clinics (i.e. Frontier Family Medical Clinic and St Luke’s Hospital Outpatient Clinic).

### Intervention: the NUHS-RHS RSC program

As determined by clinical judgement, patients with one or more chronic illnesses who were deemed stable and no longer required specialists’ care were referred for the program by specialists from NUH SOCs. Upon receiving referrals from the specialists, care coordinators (CCs) explained reasons for change of care providers and location and provided details on services available, fees and contact details of the PCC clinics. This information was given to help patients make informed decisions on their enrollment. For those who agreed, a referral letter was provided in preparation for the discharge and consolidation of care was initiated. CCs assisted in the coordination of initial appointments with PCC clinics and provided option for expedited access to the SOCs when needed. At the PCC clinics, family physicians managed the enrolled patient either through a ‘shared care’ model, i.e. in combination with relevant specialist physicians but at reduced frequency of visits, or a ‘fully discharged to primary care’ model, i.e. fully managed by the family physician. Supported by a shared EMR, a team of healthcare workers (i.e. CC, nurse, pharmacists, dietitians and psychologists), regular multi-disciplinary case conferences and training by specialists, providers at the PCC clinics work together with hospital-based providers to ensure care continuity and deliver people-centred integrated care.

#### Conceptual model

Guided by the modified Conceptual Framework of Implementation Fidelity (CFIF) [26], data was collected using multiple methods to examine adherence and moderating factors affecting fidelity. The framework proposes that the level of fidelity is influenced by moderating factors, including participant responsiveness, program complexity, comprehensiveness of policy description, strategies to facilitate implementation, quality of delivery, recruitment and context, which are often inter-connected to each other. It emphasizes the need to evaluate both implementation fidelity and the moderating factors concurrently.

Considering the nature of the program that was not time-bound, we focused on assessing program content and coverage as the measures of adherence. Content was examined using non-participatory observations and reviews of medical records whereas coverage was assessed through analysis of program administrative database. Moderating factors influencing fidelity were then examined using semi-structured interviews with healthcare providers (i.e. physicians, nurses, allied health professionals, managers and CCs). As there was no benchmarking available within the Singapore context to assess quality of delivery and comprehensiveness of policy, these two dimensions were not assessed.

### Data collection

#### Data sources

Table 1 summarizes various data sources used in this evaluation. A convenience sampling of eleven non-participatory observations of patients who were referred for RSC introductory sessions with care coordinators and agreeable to be observed were conducted. For pragmatic reasons, study team members worked closely with care coordinators and relied on their recommendations for the observations. A random sample of medical records of 30 patients enrolled in the program across different specialties and had experienced the program more than 6 months were reviewed to assess adherence (content). Thematic saturation was used to determine the eventual sample size for both observations and review of medical records. 29 reviews of medical records were finally included in the analysis. One data point was excluded due to poor documentation. In addition, program database containing patient enrollment information was examined to assess program coverage. In evaluating moderating factors influencing the program, 25 healthcare providers including CCs, managers, and physicians involved in the implementation of the program were interviewed.

**Table 1:** Data sources

	Specific component	Data sources	Nature of data
Adherence	Content	Ethnographic observations	New (collected as part of evaluation)
		Medical records	Existing (routinely collected as part of program)
	Coverage	Program database	Existing (routinely collected as part of program)
Moderating factors	Participant responsiveness	Semi structure interviews with healthcare providers involved in the program	New (collected as part of evaluation)
	Complexity of Program		
	Facilitating strategies		
	Recruitment		
	Context		

### Procedures

#### Assessment of adherence (content)

Initially, a list of intended activities, as specified in the program document and verified through conversations with program managers, was summarized and used as an assessment checklist. Each activity included in the checklist was explicitly defined to facilitate assessment of program adherence (content). These intended activities included: (i) referral of suitable patients at the SOCs by specialists, (ii) consolidation of care under one dedicated physician, (iii) stratification of care and care location according to patients’ needs and preferences, (iv) preparation of transition from SOCs to other appropriate sites by CCs (patient education, appointment management, and provision of referral letters), (v) support for PCC physician-in-charge by a team of hospital based healthcare providers, and (vi) multi-disciplinary case conferences to discuss and make changes to patient’s care plans.

Two study team members (PS and LXY) conducted non-participatory observations of the information sessions (the initial activity of the program) conducted by CCs. In every session of observation, content of care delivery was observed. Furthermore, medical records of patients enrolled in the programs were reviewed by PS, EKX, and MN to provide a comprehensive picture of the content of the program. Detailed notes were taken both during observations and with reviews of medical records.

#### Assessment of adherence (coverage)

A consolidated program database containing records of patients who were enrolled in the program and were eligible but eventually declined enrolment in the years 2014–2017 was used. Data within the database were collected as part of the routine administration of the program by the program manager. Data were checked, duplicates were removed, and data were streamlined according to the unique patient identification number for ease of analysis.

#### Assessment of moderating factors influencing implementation fidelity

A convenience sample of healthcare providers involved in the planning, development, and implementation of the program was recruited in the study. Invitation emails were initially sent out to recruit study participants from a contact list of all healthcare providers obtained from the RSC program manager. Only those who responded to the email invitations (52%) and agreed to be audio-recorded were interviewed by the study team member(s). Thematic saturation was used to determine the eventual sample size.

An interview guide (Additional file 1) was developed and used to assess moderating factors which may have facilitated or hampered the implementation of the program. Two qualitative researchers (MN and PS) conducted the interviews that lasted between 45 and 90 min. In ensuring the quality of data collected through interviews, PS and MN routinely reflected on interview experiences and discussed data as they were being collected. Interview questions were revised based on emerging understandings and identification of key knowledge gaps gathered from prior interviews.

### Data analysis

#### Assessment of adherence (content)

After achieving inter-rater consistency, MN and PS independently scored respective program components as “yes” if it was conducted and “no” if it was not. Level of adherence for each specific program component was then calculated as a percentage of number of cases (observation or record) in which the component was scored as “yes” over the total number of observations conducted and medical records reviewed. Referencing from what was previously used [26, 27], 80–100% adherence was classified as “high”, 51–79% as ‘moderate’ and 0–50% as ‘low’ fidelity.

#### Assessment of adherence (coverage)

Coverage was tabulated as the proportion of patients who enrolled in the program over the total number of patients who were offered the program by the CCs. The database showed that some patients who agreed to enroll initially ended up withdrawing from the program. As this study was interested in the overall coverage of the program, we considered those patients as “enrolled”. Moreover, to understand the evolution related to the program, we examined coverage over the years since the initiation of the program.

#### Assessment of moderating factors influencing implementation fidelity

All interviews were audio-recorded and transcribed verbatim. Each transcript was checked against recordings for accuracy. Then, MN and PS read and coded the transcripts independently using ATLAS.ti version 7. A deductive analysis was adopted to code units of data according to the modified CFIF [26]. We further classified contextual factors under providers, organizational, and system levels. During analysis period, MN and PS met regularly to discuss and confirm emerging themes identified in the data. Re-analysis was conducted if discrepancy was identified. After which, findings were shared with the wider research team and study participants for scrutiny and verification to establish the trustworthiness of findings.

#### Data triangulation

Quantitative and qualitative data collected from the various sources were given equal weightage, analysed, and merged at analysis stage guided by the modified version of the CFIF by MN and PS. Quantitative and qualitative data was mixed for the purpose of illustrating a more complete understanding of the topic being studied [28]. Thus, at interpretation and reporting level, data were integrated through the narrative approach. An information matrix was developed to relate quantitative adherence data to qualitative data on moderating factors in which qualitative themes were used to explain quantitative data. Interpretation was then shared again with the wider research team for scrutiny, discussion, and verification.

## Results

### Adherence

As illustrated in Table 2, the majority (4 out of 6) of program components were implemented with low fidelity. Stratification of care and preparation of transition from SOCs to PCC clinics by CC were found to be implemented with high fidelity.

**Table 2:** Implementation fidelity of intervention components and moderating factors affecting fidelity

Component of Adherence	Intervention Component	% of adherence	Level of implementation fidelity	Moderating factors affecting fidelity
Content	Referral of suitable patients by specialists at the SOCs	25	Low	• Providers’ responsiveness • Context (providers) • Context (organizational) • Context (system)
	Consolidation of care under one dedicated physician	0	Low	• Complexity of program; • Context (organizational)
	Stratification of care according to patients’ needs (simple conditions were referred to primary care and more complex were referred to internal medicine specialists)	100	High	• Facilitating strategies
	Preparation of transition from SOCs to other appropriate sites through patient education, appointment management and provision of referral memo by CC	90	High	• Facilitating strategies
	Support of PCC providers by a team of hospital based providers (specialists, CC, nurse, and AHP: dietician, medical social worker, psychologist, and pharmacist)	20	Low	• Context (organizational)
	Multi-disciplinary case conferences involving specialists, PCC providers and AHP to refine care plans for patients	2.5	Low	• Context (organizational) • Context (system)

*SOC* specialist outpatient clinic, *CC* care coordinator, *PCC* primary and community care, *AHP* allied health professionals

### Coverage

**Fig. 3:**
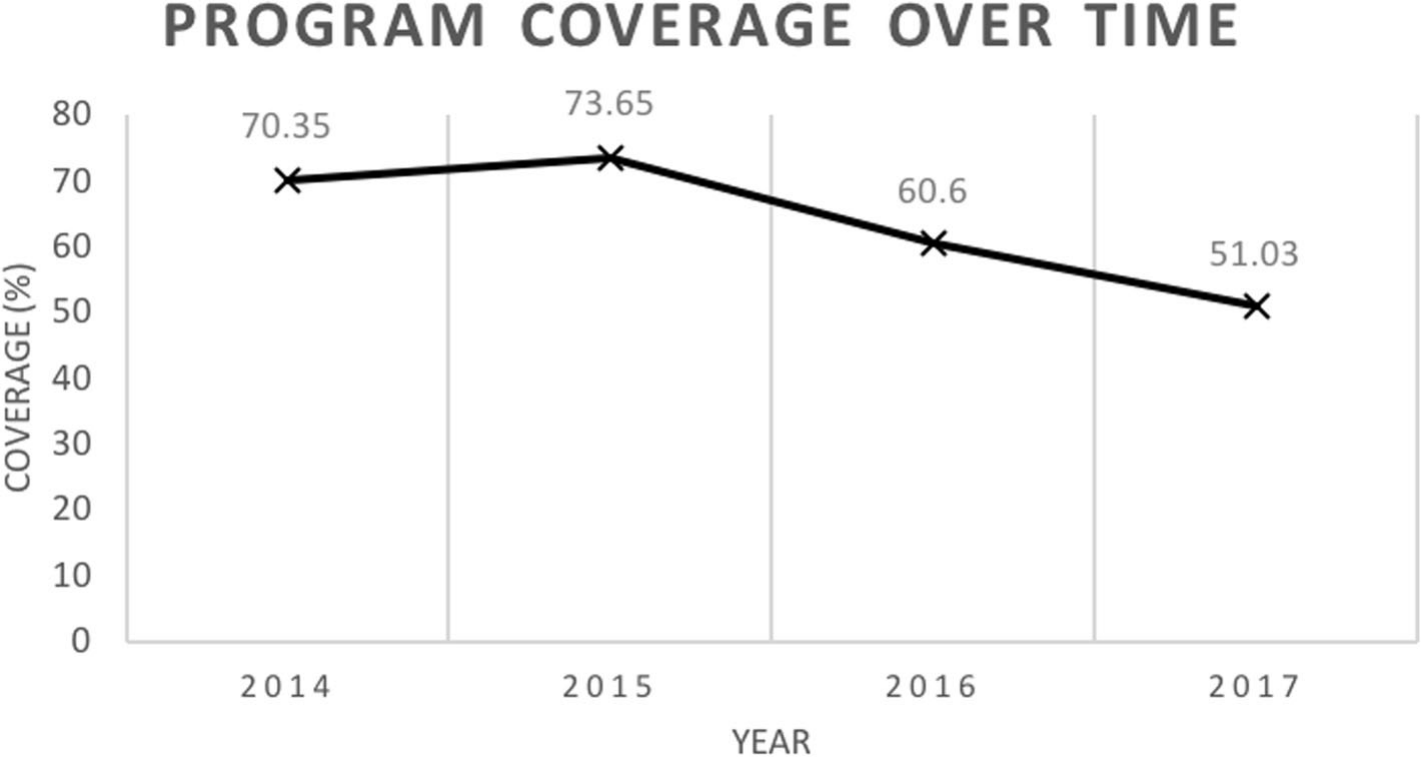
NUHS-RHS RSC program coverage over time

The RSC program database showed that a total 9112 unique suitable patients were referred to the program since program initiation. Out of which, 3032 (33.3%) declined to be enrolled. Since the initiation of the program, decreasing coverage was observed (Fig. 3).

### Moderating factors

Key themes of moderating factors and their corresponding exemplary quotes found in the study were described in Table 3.

**Table 3:** Key themes and exemplary quotes

Moderating Factors	Themes	Exemplary quotes
Participant Responsiveness	Lack of confidence in PCC providers among specialists	*“Sometimes, specialists are worried - because they are family physicians, and that these diseases are not common like diabetes or hypertension - whether they are able to handle. Although we try to move, but the apprehension of the doctors also are there. Due to the hassle of moving, they rather see them because at the end of the day, it’s only (every) four months or six months down the road. So these are the challenges that we face” (Care coordinator)*
	Limited understanding of the program	*“(The specialists think that) “I discharge (patients), who do I discharge them to? If I discharge them to the black hole ... I don’t know these people, I don’t know the people in the polyclinic, I don’t know the primary care doctor, so if I discharge them to the black hole, anything can happen to my patient.” (Family physician)*
	Ethical Dilemma: conflict between ethos and program goals	*“I mean that cost gradient is definitely not working out for patients and sometimes I wonder, am I doing the right thing for patients.” (Program manager)* *“If the purpose was to do what right siting describes, which is to move people with lower needs out to a place where there is less care, I don’t find that meaningful, it’s not something I would not do as a human being. I get up in the morning, if that was my job, I rather do something else.” (Specialist)*
	Negative feedback about PCC from users	*“When patients come back with feedback of not having enough medicines, service attitudes, they are also etched into the specialists’ mind because some patients may be with our consultants for eight to fifteen years already. If they discharge very familiar patients to primary care providers and they come back with poor feedbacks, it is not so good for their rapport.” (Care coordinator)*
Complexity of program	Evolving dynamic of collaboration	*“The relationship changed, we weren’t as deeply engaged as we were before, the team turned over as well, I mean X stepped down as medical director of the center and handed over to Y, who was a good guy! But it’s not exactly the same team anymore, I just get a sense that actually if all of the care was reverted back to the whole model, the passion for patient-centered care has to some extent died away.” (Specialist)*
	Mismatch between program goals and current healthcare financing	*“I wasn’t clear in the direction of the program, I thought if you want to right-site to primary care partners, but yet you’re making SOCs even cheaper?” (Program manager)*
Facilitating Strategies	Synergistic partnership between collaborators	*“I think we found a very synergistic partnership in NUH and us when we decided that we want to bring the care of patients to the next level, we just say shoot for the stars, and do the best that we can just to make sure that the patient is really in the center. And then all this fragmentation of care is being reduced” (Family physician)*
	Training of the PCC providers by the SOC specialists	*“One of the specialists went to the PCC on a regular basis, once a month to discuss cases. This was especially important in the first year, where the doctors there were still getting used to the idea of managing patients with more complex conditions who have been recently admitted in a tertiary hospital. So we just look at them to gain the confidence and comfort level in managing some of the patients. But this program has been running for a few years, so training is less frequent now as the need is (assumed) to be not so strong.” (Specialist)*
	Support system: care coordinators	*“It’s crucial for the care coordinators to be there … Care coordinator must be part of the clinical team, for them to be familiar … We found that the most effective way is the team having one care coordinator and then outflow through that.” (Care Coordinator)* *“I think having a care coordinator to explain things to patients is very helpful because usually clinicians can be very busy and rightfully the patients have a lot of questions to ask to get re-assured so they feel safe enough to get discharged.” (Specialist)*
	Support system: protocol for providers	*“We have a systemic protocol for recruitment … Once we identify the patient, we just pass over to the clinic assistant and the clinic assistant will immediately call the care coordinator, so the coordinator will write down the patient’s needs. If they are free, they can see on the same day. If they are not, they will give them a call and arrange for a meeting.” (Specialist)*
	Support system: shared EMR	*“With the same hospital system, they could see everything, they were able to look at the results, our train of thought, the way we have managed the patient so far. When the patient gets discharged to us, we could also see, knowingly or unknowingly, what the specialist has been thinking about and going through, the thought process in managing this patient … It gives us a better understanding of a patient’s condition and ammunition to advise the patient correctly.” (Family physician)*
	Support system: shared hospital pharmacy	*“We gave up the pharmacy revenue which constitute usually a large percentage of the primary care physician’s revenue. We gave that up because we know that is going to be a plus for the patients, they are going to get subsidized drugs … cheap drugs, and they are going to take the drugs every day. So that takes off a chunk of our revenue.” (Family physician)*
	Care model	*“The program helps because the greater access in the community will help the patient come and see us. If there’s any problem, we will escalate back to NUH.” (Family physician)* *“I realize the good points of the program is … that the time is more flexible. Even let’s say after working hour(s). You can just walk in. It’s not like the SOC (where) we don’t accept walk in” (Care coordinator)*
Recruitment	Reduction in the number of suitable patients	*“I think primarily because over the years the number of patients that considerably could be placed out from the existing pool has been exhausted to some extent … It is the new patients who are entering the sub-specialty clinics.” (Specialist)*
	Insignificant cost gradient between hospitals and PCC	*“The main barrier to recruitment right now is the specialists buying into this model and sending their patients (out). I feel that is the biggest barrier, because of (the patients’) trust and confidence on the doctors (it is hard to) let go of physician-patient relationship that was built for years.” (Specialist)* *“Five years ago, when patients come to see a specialist at the hospital, they pay $25–27 to see a specialist in the hospital. When you go to see the GP, you pay the same amount but you are only going to see a GP so in terms of value for money, I think most people would say they would prefer to come and see a specialist.”(Specialist)*
Context Providers’ level	Lower motivation among hospital providers	*“Now that the load in the clinic is more manageable, then doctors will sometimes forget about this program, they’ll just manage it day to day.” (Program manager)*
	High workload of hospital providers	*“It is not that the doctors don’t want to get more involved, but we just don’t have the time and we probably cannot pull out the information off hand. And most of the time, patients need time to sit down, think about it, discuss with their family members, have the information leaflets to think about it and compare and weigh things in their mind. So I think that’s something that we don’t have time to do at our level.” (Specialist)*
Context Organizational level:	Lack of program reinforcement	*“There are guidelines and criteria for seeing a specialist and discharge. If the patient has met the discharge criteria and we want to discharge, but no one enforces. No one audits.” (Specialist)*
	Competing agenda	*“It has been tapering, we reached a peak about a year or two ago. We are dependent on referral letters from NUH and the numbers have been going down …*. *The understanding was, it’s also partly because the awareness of our center is weaning a little bit. I think we are no longer the flavor of the month. (Family Physician)*
	Organizational disincentive	*“The hospital cut our headcount on the basis of the number of patients that we see in the outpatient clinic, so it was counterintuitive, to any right-siting program.” (Specialist)*
Context System level:	Limited capability within the PCC clinics	*“Family physicians have been practicing for decades in this comfort zone, seeing patients with simple conditions, and suddenly you’re managing patients with rheumatoid arthritis, Parkinson’s patients who you’ve never managed before in your life. So that will make some doctors uncomfortable. (Family physician)*
		*“When a patient went to see a doctor at the PCC clinic and doctor said “Oh I’ve never seen this condition before, the last I read about it was in medical school, you know how rare it is?” So obviously the patients were quite shocked when they hear that. And then they feel that they don’t have that assurance and or confidence, and they come back.” (Care coordinator)*
	Limited capacity at the PCC clinics	*“After RSC program has (been implemented), we received feedback that one of the PCC clinic has reached their capacity at one point of time.” (Program manager)*
	Fragmentation in funding: disease-centric reimbursement	*“Funding here is unfortunately on a per consult basis, not on a capitated basis, making things challenging. This is because generally patients with multiple specialty follow-ups require more time to sort through their problems, every time they see us. If I spend more time on these patients and there’s actually less time to see more patients to generate revenue for our center.” (Family physician)*
	Fragmentation in funding across care settings	*“You can’t have a fragmented funding when you talk about integrated care. You must treat primary care and the hospital as a whole, and then you must fund both as a block. The hospital now have to make sure that the funding of primary care is well-supported to make sure the patients are cared for in a good way in the community so that they do not fall back into the hospital, which will cost even more.” (Family Physician)*
	Mismatch between providers’ reimbursement and program goals	*“My bonus and performance depends on my workload. If I want to play the rules, I want to make sure that my clinic is always full. I’ll (repeatedly see the same patient). I can see 25 patients on their repeat visit. My workload will look very good so my bonus should be more and I don’t have to deal with the complicated new cases; life will be easier for me.” (Specialist)*

#### Participant responsiveness

##### Providers’ perception of the program influenced their level of engagement

Different levels of responsiveness were observed among specialists, depending on how they perceived the program. Expectedly, those who had positive views of the program saw it as a strategy to overcome issues related to congestion within the SOCs and were better well-engaged by the program than those who were not. Varied perceptions were influenced predominantly by specialists’ understanding about the program and their trust levels of the PCC providers. Specialists were accustomed to working within their disease specialties, with services typically charged to patients based on disease, service, and provider type within the respective speciality clinics. Such disease-centric reimbursement limited the specialists’ understanding of the program and familiarity with how the program ran. In addition, with disease-centric clinic set-up and little coordination among specialities, fragmentation of care services persisted within the acute care setting. Therefore, the concept of “one-stop shop” was considered different by providers as patients with multimorbidity commonly had to shuttle between different clinics for their treatments. Scepticism among hospital-based providers about the collaboration was also reported, likely due to little experience working with others outside of the hospitals.

##### Providers’ experiences of the program determined their level of responsiveness

Among those who were initially supportive of the program, ethical dilemma surrounding the benefits of the program to patients led to diminishing responsiveness. Providers had to balance their perceived benefits of the program to patients with the program mission of referring as many patients as possible out of the hospital. This is because the program required users to pay less “out of pocket” cost for the services utilized within the community compared to when services were utilized in the hospitals due to the large subsidy provided by government for the use of public hospital based care. Furthermore, fee incurred within the hospital can be paid through Medisave (compulsory medical saving account).

Some specialists also felt *conflicted* as they perceived PCC providers to be providing “inferior” quality of care compared to the hospitals, and described the program to be “against the specialists’ ethos of patient care”.

Some patients returned to the specialists due to suboptimal experience in the PCC clinics related to providers’ capability and service quality. Poor feedback from patients adversely affected the hospital providers’ confidence of PCC which was then subsequently translated into lower responsiveness to the program.

#### Complexity of program

Despite the system-wide emphasis for collaboration, our findings showed slow progression in collaboration within the program. Dynamics of the collaborations was described to change over the years in an unpredicted manner. Level of engagement of various stakeholders fluctuated with changes in the team structure and leadership. Consequently, it adversely affected the passion for and commitment to the program among stakeholders.

Even with best efforts to explain and convince suitable patients to enroll in the program, it was challenging to shift chronic care from the hospital to the community. Existing healthcare financing was reported to contradict with the goals of the program. Heavily subsidized hospital care was said to inevitably shape patients’ preference for hospital care. However, despite feedback from staff about the mismatch, the problem persisted and caused great frustration among providers.

#### Facilitating strategies

Synergistic partnership, training of PCC providers by specialists, and supportive structures including CCs, guiding protocol for the providers, shared electronic medical records (EMR), and shared pharmacy were found to facilitate program implementation. With the ultimate goal of providing people-centered care, synergistic partnership was fostered to enable collaboration. The common goal united stakeholders between the NUH and PCC providers. Upon which, an appropriate model of care and new workflow was developed, resources were invested, and collaborative working relationships were established.

As part of the program, training for PCC providers by the specialists were initiated to equip them with necessary knowledge and confidence. Specialists organized seminars to discuss about management of chronic diseases and visited the PCC clinics to see patients and/or discuss cases. These activities also helped to build rapport between providers across healthcare settings, which in turn facilitated efficient and effective communication. However, training was reportedly conducted less frequently as the program was assumed to have reached a stable stage. With decreased contact time with specialists, PCC providers felt less supported and confident in managing more complicated conditions. Thus, patients were noticeably referred back to the SOCs more frequently than before. At the same time, the PCC providers’ decreasing confidence adversely influenced patients’ confidence in PCC, leading to an increased number of patients’ voluntary returns to the SOCs.

In supporting the program, CCs were noted to play important roles as care integrators to connect between healthcare providers from varied SOCs and healthcare users (patients and caregivers) in care consolidation and to prepare for and support the care transition. Some CCs also went the extra mile to review the patient list in an effort to assist the specialists in identifying the stable patients suitable to be discharged/transitioned, thus enhancing the patient recruitment numbers. Busy specialists regarded the coordination by the CCs to be useful and was key to the success of implementation of the program.

Guiding protocols describing selection criteria and main steps for program delivery were developed at the beginning of the program. It helped the healthcare providers in managing patients’ complex conditions according to the intended model of care in a standardized manner. Furthermore, it also included an escalation workflow to be used in the event of unexpected deterioration of patients’ conditions. The RSC program was favored by some providers as the model of care was said to provide greater accessibility for patients because of PCC clinics’ convenient locations within the community and their flexible opening hours and acceptance of “walk-ins”.

Shared infrastructures including the common EMR and shared pharmacy adopted as part of the program were identified to be pivotal for the success of the program. All healthcare providers involved were granted access to the common EMR which they would not have if they were not part of the program. The common EMR functioned to systematically consolidate medical information and allowed sharing of information between actors across disciplines and care settings, thus facilitating care continuity. Hospital-based CCs were also given access to arrange for appointments with the PCC providers, streamlining the process for patients so as to reduce barrier to enrollment.

Nonetheless, shared EMR was found to be not fully compatible to the needs of PCC as it was created for hospital-based care and processes. Therefore, in addition to the shared EMR, PCC providers had to use their own system for billing purposes and used the shared EMR solely for clinical information. This created unhappiness within the PCC providers as it created unnecessary workload for their employees.

To lower the barrier for program enrollment, satellite hospital pharmacies were established within PCC clinics to provide specialists drugs at a similar subsidized hospital rate. This was found to be particularly well received by patients as they could obtain their medications easily near their homes. Nevertheless, the strategy substantially reduced PCC clinics’ revenues from dispensing medications. Even though PCC clinics received funding to support the RSC program, the PCC providers were concerned about the sustainability of their clinics with the reduction of revenues from medication dispensing.

### Recruitment

The number of suitable patients was found to decline over time. This was partly because most suitable stable patients were discharged in the early phase of the program. Specialists’ “buy-in”, a reflection of their responsiveness to the program, was acknowledged to be the main determining factor behind decreasing recruitment numbers. As patients who were identified and sent directly by the specialists were more likely to agree to enroll in the program compared to those referred by other sources, decreasing referral from specialists expectedly resulted in lower recruitment number.

With insignificant cost gradient between the hospital SOCs and PCC clinics, seeing a specialist in the hospital cost patients the same or cheaper than having their care managed by the PCC providers. This was considered “not of value” for patients. Consequently, despite having greater access to PCC, patients refused to move out of the SOCs. This in turn reduced providers’ motivation to promote the program as they perceived that the program was “not value for money” for patients and hence not “worth the effort”.

#### Context

At the providers’ level, lesser congestion in SOCs due to the transfer of patients in the initial phase of the program lowered motivations among the specialists to actively recruit patients for the program. Furthermore, some specialists regarded the introduction of the program to the patients to be time consuming. They would rather monitor existing patients annually or bi-annually than spend the extra amount of time explaining about the program and convincing patients to see the CC to learn about the program.

At the organizational level, sharing about program at the beginning through roadshows by representatives from PCC had prompted active participation of providers. Nonetheless, without continuous organizational reinforcement, specialists lost sight of the program, resulting in lower responsiveness and subsequently lower recruitment numbers over time.

Besides the RSC program, the NUHS RHS Planning Office was reported to be heavily involved in the implementation of other existing programs and development of new programs aimed at fostering integrated care within its geographic region. Given the limited resources within the NUHS RHS office to manage numerous projects under its purview, the RSC program’s visibility was perceived to have been diluted by uncertainty in its future directions relative to other programs.

While the initial decrease in patient load within the SOCs was well received by providers, a reduction in resources that came with the decrease in the number of patients managed at the SOCs discouraged further referral of patients out of the SOCs. There was a constant pressure within the SOCs to keep patient volume relatively high so as to avoid removal of resources.

At the system level, fragmentation in care capabilities among providers were also observed. CCs could not consolidate care of patients under one physician after discharge from the hospital as PCC providers lacked confidence and capability to continue management of multi-morbidity. This dampened patients’ confidence of the PCC providers.

Limited capacity at the PCC clinics also prevented the clinics to take in more patients as they were overwhelmed with the high number of patients. The disease-centric reimbursement created fragmentation of the funding across the different care settings and was found to impede the implementation of the program. Without a mechanism to pool charges across services and sectors, it was challenging to convince patients to agree to enrol in the program given the long withstanding perception of inferiority of primary and community care in Singapore.

Likewise, fragmentation in funding segregated the healthcare system, making integration of care across separate entities difficult. Since healthcare funding was largely concentrated within the hospitals, there was limited resources available within the PCC clinics to raise their capacity and capability to manage the rising load of individuals with multi-morbidity. As a result, the introduction of new models of care like the RSC program was difficult as incentives were not aligned across patients and providers.

Providers’ motivation was also found to be influenced by how they were reimbursed. Typically, productivity of providers was measured by the number of patients they managed and they were reimbursed by volume regardless of complexity. With this model of reimbursement, it was not profitable for the specialists to refer stable patients to be discharged to free up slots for intake of new patients/complex cases. New and/or complex cases were reported to usually take up more time for consultation and adversely affect the volume of patients the specialists can see. Therefore, it was considered counterintuitive for specialists to refer their patients to the program. Instead, specialists chose to retain their existing patients who were easier to manage so as to maintain a high volume within their clinics.

#### Interpretation

**Fig. 4:**
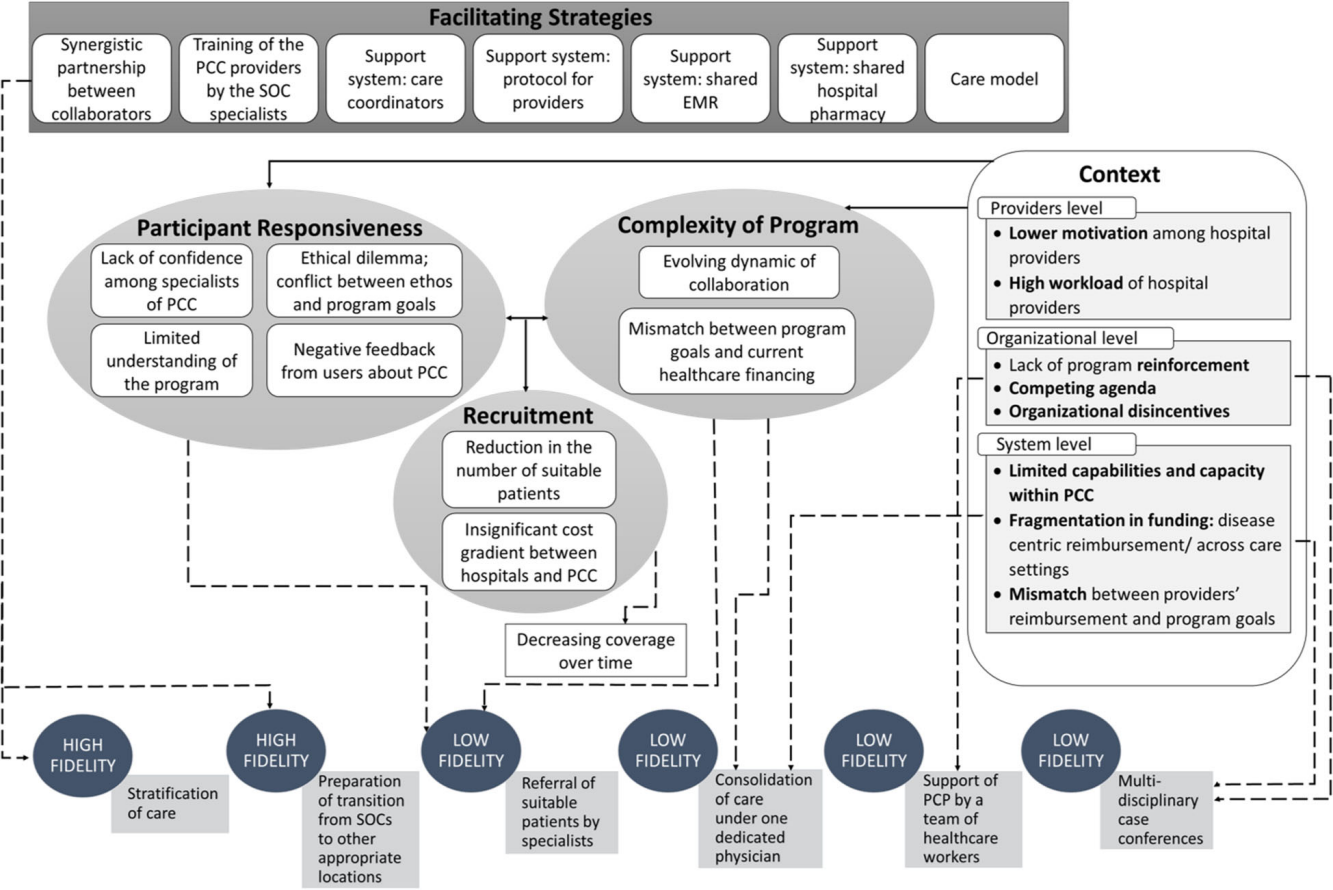
Implementation fidelity of the NUHS-RHS RSC program and moderating factors influencing implementation

Using a narrative approach for data merging and interpretation, Fig. 4 illustrated our study findings and revealed the interrelated influence of moderating factors specified by the modified CFIF on the adherence of the program.

## Discussion

Using the modified version of CFIF [29], we evaluated the implementation fidelity of the NUHS-RHS RSC program, a collaboration between a tertiary hospital and the PCC clinics in the western part of Singapore. Our findings revealed variations in the level of implementation fidelity of various program components, which were moderated by factors related to the providers, organizations involved, and the healthcare system.

High implementation fidelity found for preparation for transition and stratification of care and care locations can be attributed to adequate facilitation by CCs and supportive systems, particularly program guidelines and shared IT system. Preparing patients to transition from hospitals to PCC was new for most CCs involved in the program and detailed protocols gave step-by-step guidance for CCs to standardize information and confidently relay them. This is consistent with another study which suggested that detailed protocols facilitated implementation of complex healthcare programs [18]. Moreover, access to the shared EMR provided CCs with a comprehensive view of the entire patients’ healthcare journey, providing CCs with relevant information to develop appropriate care plans and whom they can work with to manage care for a patient. In other contexts, EMR had also been shown to enhance communication capacity and information flow by linking consumers and providers across the continuum of care [30].

We found low implementation fidelity for referral of suitable patients by specialists and decreasing coverage over time to be related to the complexity of program and diminishing providers’ responsiveness. Similar to what was experienced in the implementation of the PCMH across the USA [31], we found that interactions between various actors shaped the behaviour of actors and the program as a whole. Typical of a complex adaptive system [32, 33], the dynamic interaction between stakeholders within the program rendered the program complicated. Dynamics within the collaboration was reportedly volatile and dependent on leadership, team structure, and model of reimbursement. Frustrating persistent mismatch between program goal and existing healthcare financing was also found to contribute to program complexity and negatively affecting providers’ motivation. Additionally, diminishing providers’ responsiveness due to the lack of knowledge about the program, ethical dilemma, and fear of losing continuity of care were shown to deter referrals from specialists. This was accentuated by reports of suboptimal experiences at the PCC clinics by the healthcare users which further discouraged referral. Furthermore, traditional “fee-for-service” model that incentivizes providers based on the volume of patients dissuaded referral of patients into the program. Likewise, disease-centric reimbursement distorted providers’ perception of the importance of holistic care and value of the RSC program. This lack of organizational financial incentives was similarly found to be the key barrier for collaboration between hospital and primary care in the USA [34].

Consolidation of care was restricted mainly by the complexity of patients’ needs accompanied by limited capacity and capability of the PCC clinics to manage some conditions. Given varied severity levels of patients’ conditions, it was often not feasible to consolidate care under one physician. Even when consolidation of care was deemed suitable for a particular patient, specialists’ approval, and capability and capacity of the PCC clinics determined whether care was eventually consolidated. With diminishing providers’ responsiveness, lesser consolidation of care could be expected as specialists prefer to keep their patients in their clinics.

Low implementation fidelity found for support of PCC providers by hospital-based healthcare providers and multidisciplinary case discussion could be attributed to reduction in resources and organizational reinforcement. Reduction in resources within the SOCs lowered the capacity of the specialists to spend “additional” time to support the PCC providers or participate in multidisciplinary case discussion. Furthermore, multidisciplinary collaboration was not sufficiently rewarded as providers were reimbursed based on the number of patients without taking into account the complexity of the cases and the amount of time spent on case discussions. Without appropriate incentives to motivate providers, it was not surprising that the “additional” activities such as supporting the PCC providers and multidisciplinary case discussions were not prioritized. Sequentially, these adversely affected stakeholders’ rapport that may eventually result in suboptimal care quality.

### Recommendations

Based on our study findings, we propose several recommendations for consideration when designing and implementing a program to shift care from hospital to the community as part of the strategy to integrate care. First, efforts should be put in place to improve providers’ responsiveness to program implementation. Second, improving the quality of collaboration among actors should be prioritized. Third, incentives should be aligned to the program’s goals. Finally, active engagement of patients in the design and development of the program should also be explored.

In enhancing providers’ responsiveness to the program, efforts should be put into improving providers’ awareness of the program and addressing mistrust between providers in the two settings. Roadshows and introduction of program at various SOCs which worked well can be resumed. Confidence among specialists can also be regained by the sharing of positive outcomes as a form of positive reinforcement. It may be useful to combine this sharing with implementation or reinforcement of guidelines, as the combination of both has been found to be more effective than either alone [35].

The RSC program can be incorporated as a core clinical program to be introduced early in a patient’s healthcare journey. This is expected to not only emphasize the program to providers but serve as additional positive reinforcement to boost patient enrollment. A previous local study suggested that patients who had been seeing specialists for less than 2 years were more likely to agree to be transferred out of the SOCs [20].

In order to improve collaboration between different stakeholders, the complex adaptive system perspective can be adopted to inform understanding of relationships and dependencies between different parts of the program [36]. This needs to account for the lack of homogeneity and conformity, and the difficulties in designing an optimal system in advance, given the many moving parts. Building on the insights gathered, continual and active engagement of different actors through networking sessions not to homogenize perspectives but building on an understanding of common goals, roles, commitment, and strengths should be prioritized [37].

Appropriate incentives (financial and non-financial) aligned to the program’s goals should be introduced system-wide in support of the program and to lower adoption barrier by providers and patients. A mechanism which pool charges across services and allow subsidies to follow patients could be explored to make PCC more appealing to patients. Instead of the traditional “fee-for-service” model, “fee-for-performance/complexity” can be explored to pay providers and organizations based on the complexity of illness and improvements of patient outcomes achieved [38]. This would not only enable implementation of this program but will also facilitate the national priority of providing people-centred care in the community.

Even though this study did not evaluate patients’ perspectives of the RSC program, a previous study that examined patients’ experiences with the RSC program showed that there was a lack of understanding of the comparative advantage of community-based care and its contribution to long-term health outcomes [24]. For this reason, the authors proposed for greater patient engagement. This is consistent with the growing body of literature which suggests that engaging patients can lead to improved healthcare outcomes, quality of care and care experiences [39], health service utilization [40], and lowered healthcare related cost [41]. Furthermore, successful patient engagement through co-designing has been shown to result in care improvement, cultural change within organization, meaningful collaboration, and mutual learning [42]. Learning from these, a regular patient engagement with the goal of improving the design of the program should be explored for further development of the RSC program. Since patients in Singapore are largely passive [43], it would be important to activate patients to participate by offering flexibility in the levels of engagement and approach, and including a reward mechanism to incentivize participation. Institutional commitment to prioritize patient engagement should also be emphasized.

### Strengths and limitations

In the context of Singapore, where most prior evaluation of similar programs mainly focused on assessing the effectiveness [19, 23], our study provided important insights to explain the outcomes of the program and facilitate improvements in shifting care from hospitals to community. We used a validated framework, the modified CFIF, which has been used to assess implementation fidelity of complex healthcare programs [29, 43, 44]. This allowed the identification of specific areas for improvements and comparison with other studies that had used the same framework. However, due to the design of the program, adherence in terms of dose and frequency were not examined. While collecting data, we observed limited documentation after discharge from the hospitals, making quantitative assessment of program activities after discharge difficult. For comprehensiveness, we had to complement the lack of information with qualitative data. We were also unable to evaluate quality of care and comprehensiveness of policy due to the lack of program level data and benchmarking. Nonetheless, we managed to tease out important components which are crucial to answer our research questions.

Collection of data alongside the implementation of the program ensured that data reflected the actual circumstances surrounding the program, thereby provided confidence about the credibility of the study. However, due to the cross-sectional nature of the study, we did not take into account the changes of the program over time. Therefore, the effects of the evolution on implementation fidelity of the program could not be explicitly highlighted. This points for a future longitudinal investigations to better understand how implementation of the program changes over time and its impact on outcomes. In order to do so, an increase in grant funding of implementation science research is needed.

In this study, pooled analysis was conducted instead of stratified analysis by the type of partner PCC clinics and only common key findings are included in this manuscript for overall representation of the program. While we are confident that findings are to a great part generalizable, given that various PCC clinics were involved in this program, it may be value-adding to further analyze the results according to the type of partner PCC clinics so that engagement intervention for specific PCC settings can be tailored.

## Conclusion

We found that facilitation through synergistic partnership, training of PCC providers and supportive structures (CC, program protocol, shared EMR and pharmacy) contributed to high implementation fidelity. In contrast, complexity of program, diminishing providers’ responsiveness, lack of re-inforcement, and mismatch between program goals and healthcare financing were found to make program implementation challenging. While it was acknowledged that adequate facilitation through reliable support system was essential [21], our study highlighted that functional integration alone was not sufficient for a successful program implementation. Instead, an integrated care intervention like the RSC program should be approached comprehensively from micro (patient-provider) level, meso (professional or organizational) level, and macro (system) level in order to achieve desired outcomes [45]. Similar to what was proposed for the implementation of PCMH, our study also elucidated the need to improve relationships among patients, providers, organizations and payers.

## Supplementary information

**Additional file 1.** Topic Guide for Interviews.

## Data Availability

Full transcriptions of the qualitative data gathered and analyzed are not available as they may contain quotes and identifiable information that could compromise the identity of participants. However, expanded themes are available from the corresponding author on reasonable request.

## Abbreviations

CC: Care Coordinator
CFIF: Conceptual Framework of Implementation Fidelity
EMR: Electronic Medical Record
NUH: National University Hospital
NUHS: National University Health System
PCC: Primary and Community Care
PCMH: Patient Centered Medical Home
RSC: Right-Site Care
RHS: Regional Health System
SOCs: Specialist Outpatient Clinics
USA: United States of America
WHO: World Health Organization
