# Supplementary material for: Shifting care from hospital to community, a strategy to integrate care in Singapore: process evaluation of implementation fidelity
Source: BMC Health Serv Res. 2020 May 24;20:452. doi: 10.1186/s12913-020-05263-w (PMC7245814; doi:10.1186/s12913-020-05263-w)
Supplement: Supplementary file 1 — Additional file 1. Topic Guide for Interviews. [file 12913_2020_5263_MOESM1_ESM.docx]

**Annex 1: Topic Guide for Interviews**

1. **[Adherence]**
   1. **[Coverage & Recruitment]**

- How were patients enrolled into the programs?
- What proportion of eligible patients were enrolled into the program?
- Did you face any difficulty during recruitment? What were the challenges you faced during recruitment?

**1.2 [Content]**

- To what extent were the activities implemented as planned?
  - What were the activities included as part of the program?
    - Who were involved in the activities?
    - Did you face any difficulty in the delivery of the program? If yes, please share the challenges you faced.
  - Did you involve the patients and (or) their caregivers during the programs?
    - How did they respond to the program?

1. **[Moderating Factors]**
   - What were the key success factors of the implementations of program?
   - What were the challenges faced during the implementation of the program?
   - Did you feel prepared to do what was expected of the program?
   - Were the cultural, social, organizational and political factors supportive of the implementation of right-site care program? What makes you say that?
2. Any other comments about the right-site care program?
